# **Understanding the facilitators and barriers to barcode medication administration by nursing staff using behavioural science frameworks. A mixed methods study**

**DOI:** 10.1186/s12912-023-01382-x

**Published:** 2023-10-12

**Authors:** Kate Grailey, Rabia Hussain, Elisa Wylleman, Ahmed Ezzat, Sarah Huf, Bryony Dean Franklin

**Affiliations:** 1 Centre for Health Policy, Institute of Global Health Innovation, Imperial College London, London, U.K.; 2 Imperial College Healthcare NHS Trust, London, U.K.; 3 School of Pharmacy, University College London, London, U.K.; 4 NIHR Imperial Patient Safety Translational Research Centre, London, U.K.

**Keywords:** Behavioural science, Electronic medication administration, Patient safety, Qualitative research, System Change

## Abstract

**Introduction:**

Barcode medication administration (BCMA) technology helps ensure correct medications are administered by nursing staff through scanning of patient and medication barcodes. In many hospitals scanning rates are low, limiting the potential safety benefits. We aimed to explore the barriers and facilitators to BCMA use in a London hospital.

**Methods:**

In this mixed methods study we used local quantitative data on BCMA scanning rates to identify clinically similar wards (in terms of patient acuity and workload) with different scanning rates for qualitative exploration. Interviews designed to elicit barriers to using BCMA technology were conducted with nursing staff, supported by observations of medication administration. Qualitative data were analysed inductively and a thematic framework constructed housing key themes, subsequently categorised into barriers and facilitators. To explore patient perspectives of BCMA scanning, a purposive sample of patients were also interviewed. These patient data were analysed deductively according to the thematic framework. Themes were mapped to behavioural science frameworks to further understand the behaviours involved.

**Results:**

BCMA was operational on 15 wards, with only six having medication scan rates of more than 10% of scannable doses. Of three wards selected for qualitative investigation, the lowest scan rate was 6.7%. Twenty-seven nurses and 15 patients were interviewed. Eleven key themes were identified, encompassing both barriers and facilitators to BCMA use. Barriers included poor trolley ergonomics and perceived time inefficiency. Facilitators included a streamlined process and thorough training. All nurses described BCMA as positive for patient safety. Patients described BCMA as making them “feel safer”. Behavioural science frameworks highlighted the importance of professional role and an individual’s belief in their capability.

**Conclusion:**

We present a novel exploration of facilitators and barriers to BCMA use from the viewpoint of both patients and nursing staff, highlighting a strong perception that BCMA enhances safety. Barriers were reported on both high and low usage wards, demonstrating the importance of behaviours and motivations. These findings provide a detailed understanding from which to design interventions to support behaviour change and increase BCMA use.

**Supplementary Information:**

The online version contains supplementary material available at 10.1186/s12912-023-01382-x.

## Introduction

Barcode medication administration (BCMA) technology is designed to improve medication safety during inpatient drug rounds. BCMA supports medication administration by requiring scanning of barcodes on the patient’s wristband and medication packaging by nursing staff. BCMA has been shown to improve patient safety by increasing adherence to the ‘five rights’ of medication administration (right patient, drug, dose, route and time) [1]. Implementation of BCMA has been demonstrated to reduce medication administration error rates by up to 54% [2] and create an enhanced perception of safety by nursing staff [3].

Several studies evaluating the success of BCMA implementation have highlighted challenges in achieving high scanning rates. Barriers to BCMA use can be categorised into “process issues” (such as inadequate training or negative changes to workflow), “technology concerns” and “staff resistance” [4, 5]. An ethnographic study evaluating BCMA suggested it can lead to longer medication administration rounds as a consequence of increased drug checking [6]. Conversely, another observational study exploring the impact of BCMA on nursing workflow reported that time spent on medication administration activities was unchanged, while time spent on inefficient activities (such as acknowledging physician orders) was reduced [7]. BCMA systems and software interfaces have also been described as “not user friendly” [8] and “intimidating” [9], highlighting the importance of nursing involvement in their design and implementation when aiming to achieve high scanning rates.

Much of the published literature regarding BCMA originates from the United States. These studies demonstrate typically high rates of BCMA scanning, even prior to improvement initiatives. For example, a study by Van Ornum et al. improved BCMA scanning rates from 95 to 98% [10]. The challenges faced implementing BCMA in the United Kingdom (UK) differ, likely due to differences in medication packaging, systems of medication administration and computer systems. A recent quality improvement project [11] implemented three quality improvement cycles including real-time monitoring of compliance, resulting in an increase of scan rates to 94%.

Existing research on BCMA use focuses primarily on exploring the experiences of nursing and pharmacy staff, but has also highlighted the potential impact of BCMA on nurse-patient interactions. A study using direct observation throughout the implementation of BCMA [2] in three nursing inpatient units acknowledged nursing concerns regarding patient understanding of the new system, and designed a handout to explain the process and its potential safety benefits for patients. A quality improvement project [12] built upon observations of BCMA being used in practice and noted that patients may be anxious about new technologies. The study team therefore developed a script for nurses to use with patients to introduce BCMA and allay any fears. An article by Englebright et al. [13] discusses causes of workarounds to BCMA technology, suggesting that nurses may not use BCMA at night to avoid waking/disturbing patients. The authors again promote use of a script to manage patient expectations, and advise involving patients in the process - encouraging them to “remind nurses to scan their wristbands”. Whilst the potential impact of BCMA technology on nurse-patient interactions is acknowledged, there is a gap in the current evidence with respect to directly investigating and reporting patient views on the topic.

This study therefore aimed to understand the facilitators and barriers to BCMA use experienced by nursing staff at a London NHS hospital trust with low initial uptake of the technology, additionally incorporating patient perspectives to provide a holistic evaluation of experiences of BCMA.

Specific objectives were:

1. To identify the current level of use of BCMA by nursing staff within one NHS trust and any patterns associated with its uptake.
2. To conduct a focused qualitative exploration and thematic analysis to identify common themes regarding BCMA use among nursing staff, with a focus on facilitators and barriers.
3. To utilise behavioural science frameworks to improve understanding of existing patterns of use and guide the development of any future interventions aimed at improving BCMA scanning rates.
4. To obtain a novel and holistic understanding of experiences of BCMA technology by incorporating patient perspectives.

## Methods

### Study design

This study utilised mixed methodology using an exploratory design, where qualitative methods were selected to explore and understand initial quantitative results. Local quantitative data on BCMA scanning rates were extracted and interrogated to establish the current pattern of BCMA use. Qualitative semi-structured interviews with nursing staff and patients were chosen to elucidate and understand the presence of facilitators and barriers to BCMA use. These qualitative interview data were supplemented by ethnographic observations of drug rounds.

Qualitative data were further analysed using behavioural science frameworks to enhance understanding of the behaviours arising from the identified facilitators and barriers, and identify potential foci for future intervention.

This project was deemed to be a service evaluation, with approval obtained from the NHS trust concerned (Registration no. 652 and 698).

### Study setting

First implemented at the trust in 2019, uptake of BCMA was lower than anticipated, and significantly under an initial target of 50% of scannable medication doses being scanned. The BCMA system was integrated with the trust’s electronic prescribing and medicines administration (EPMA) system to give ‘closed loop’ medication administration functionality, supporting confirmation that the correct medication is administered to the correct patient [14]. In line with most UK hospitals, nurses administered medication from original packs; the unit dose system is not used. At the time of this study, not all medication doses could be scanned. Examples of unscannable medication included any that did not have a barcode, those brought in from the patient’s home, those repackaged by the pharmacy department, and controlled drugs (which are subject to a different workflow). To administer medication using BCMA, the nurse is required to scan both the patient’s wristband, and the barcode on the medication due to be administered. The process is therefore a two-step one, and it is possible to only scan either the patient, or the medication.

As part of the implementation strategy for BCMA within the trust, new trolleys that housed a computer and lockable storage for a wide range of medications were introduced. These trolleys were selected through discussion and testing of a variety of available models with nursing staff prior to implementation.

One hospital within the trust was selected as the focus of this exploration. At the time of the study, BCMA was live on 15 of 22 inpatient wards in this hospital. We selected three target wards for qualitative exploration with different rates of BCMA scanning, aiming to select wards of the same size, layout and with similar patient demographics. Based on overall scanning rates, wards with higher BCMA usage (scanning > 10% of scannable doses), medium (5–10%) and lower usage (< 5%) were identified.

### Participant groups

Qualitative data regarding BCMA technology were to be obtained from nursing staff working on the target wards. All nurses working on the ward during the study period were eligible for inclusion, including agency staff, as this reflected the typical staffing situation at the study site. This was to ensure all barriers to BCMA use were captured, as we anticipated they might be different for different groups of staff. Patients on these wards who were likely to have been exposed to BCMA during their hospital stay were also interviewed. All inpatients on the three wards were eligible for inclusion in the study, however those who were too unwell to participate, were unable to consent / lacked capacity to participate or where language was a significant barrier were excluded.

### Data collection & analysis strategy

Quantitative data were extracted from the Trust’s local database in November 2021. These were analysed within Microsoft Excel using descriptive statistics to ascertain whether patterns were present in the timings of BCMA use, which wards were using the technology more frequently and which medications were being scanned regularly.

The qualitative interview topic guide for nursing staff was developed and informed by a literature review and discussion within the multidisciplinary team that formed the study working group (Appendix 1). The topic guide was initially piloted on nurses working in the mid-usage ward with subsequent refinement and roll out to the higher and lower usage wards.

Interviews across the 3 wards were undertaken by 5 members of the working group (RH, KG, SH, EW, AE) between December 2021 and March 2022. Prior to each interview, the nurse was provided with a brief overview of the topic and given the opportunity to ask questions. Verbal informed consent was requested prior to commencement of the interviews and they took place in private rooms within the nurse’s working environment. Detailed handwritten notes reflecting the interview content were taken during the interviews. Data obtained from qualitative interviews were analysed iteratively as the interviews progressed, allowing for refinement of the topic guide and exploration of emergent themes. Recruitment for these qualitative interviews ceased when an assessment of thematic saturation was made by two researchers (KG and RH) [15, 16].

Ethnographic observations of nurses administering patient’s prescribed medications during drug rounds were undertaken alongside qualitative interviews by one researcher (RH) in January and February 2022. The primary focus of these observations was nursing staff using BCMA to administer patient medication, incorporating events such as interruptions and distractions that may contribute to not using the technology. Again, observations were recorded as detailed handwritten notes, ensuring that behaviours surrounding BCMA use and the potential causes for these behaviours were captured. These were reviewed by two researchers (KG and RH) and analysed deductively against the thematic framework constructed during the thematic analysis of nursing interviews. These data were used to supplement the analysis of qualitative interview data.

Qualitative interview data were analysed and coded according to a thematic analysis technique [17], using an inductive approach to identify themes. The stages of the thematic analysis were as follows: familiarisation with the data set, generation of initial codes with ongoing review of the data and an evaluation of the entire data set for themes. These steps led to the construction of a thematic framework. This process was recursive, with ongoing re-review of the original data at each stage. This coding was primarily completed by one researcher (KG), with cross-coding of > 10% of the data undertaken by a second researcher (RH) to enhance credibility of the conclusions drawn and minimise potential bias. Any discrepancy in coding was resolved through discussion within the research team. Data were re-reviewed and checked to improve concordance of themes and support confirmability and dependability, with clear documentation of the analysis process. Themes and sub-themes were organised into those that represented facilitators versus barriers to BCMA use. Qualitative data obtained through observations were analysed deductively using the thematic framework generated during the thematic analysis of qualitative interview data to provide further data on the barriers and facilitators to BCMA use.

A second topic guide was developed for patient interviews (Appendix 2). Patient participants were identified through discussion with the nurse in charge (thereby ensuring patient selection was appropriate, individuals had capacity and were able to understand and converse in English). Prior to interviews, patients were provided with an introduction and explanation of the project, and verbal informed consent was obtained. These interviews were conducted by two members of the research group (RH and KG). Handwritten notes were made during these interviews, and recruitment ceased following an assessment of thematic saturation. Qualitative data from these patient interviews were analysed using a deductive approach based upon the thematic framework developed through analysis of nursing staff interviews. Again, to ensure confirmability and dependability, coding were reviewed by two researchers (RH, KG), with discrepancies discussed within the wider research team.

All qualitative themes constructed during the inductive qualitative thematic analysis of nursing interviews (and supported by patient interview and ethnographic data) were then reviewed in the context of the quantitative data obtained from each ward, with the aim of explaining the quantitative results.

#### Behavioural science frameworks

Behavioural science frameworks allow the influences on different elements of behaviour to be identified and understood. The COM-B framework identifies three essential factors that need to be present for any behaviour to occur: capability, opportunity and motivation [18]. The theoretical domains framework (TDF) [19] is a comprehensive framework incorporating individual factors that may influence behaviour (such as knowledge, intentions and goals), additionally including social and environmental factors (e.g. social influences). The framework can be used to understand the wide range of influential factors on behaviours and support the development of future behaviour change initiatives. This study utilised the TDF by mapping the facilitators and barriers identified within the qualitative work to each domain (where relevant), allowing the research team to evaluate the influences on the behaviours observed and described in interviews.

This study utilised both frameworks to comprehensively understand the behaviours associated with BCMA use, and to provide foci for future intervention. The 14 domains within the TDF were mapped to the COM-B framework in line with previous published guidance [20–22]. The thematic framework created through qualitative analysis of nursing staff interviews was then mapped to these frameworks to identify influences on behaviours, inform systematic intervention design and understand potential mechanisms of change.

### Reflexivity

RH is a research pharmacist, KG is a postgraduate researcher with a clinical background in anaesthesia, EW and AH are junior doctors, SH is a surgeon and clinical lecturer in behavioural economics in health, BDF is a research pharmacist and professor of medication safety. KG, SH and BDF all have previous experience in conduct and analysis of qualitative and mixed methods studies in the clinical environment. All authors anticipated that there would be barriers and facilitators to the use of BCMA and that these may differ between wards, but had no preconceived ideas regarding their manifestation. The team were aware of how their backgrounds may affect study design, analysis and interpretation. A reflexive position was maintained throughout to minimise the risk of bias or presumptions affecting the analysis or interpretations.

This manuscript is written in accordance with the Standards for Reporting Qualitative Research [23] (Appendix 3).

## Results

### Quantitative findings and selection of wards for qualitative exploration

The initial quantitative analysis highlighted a level of BCMA use by nursing staff that was considerably lower than an initial target of 50%. At the chosen hospital site only six wards had scan rates of more than 10% of scannable medication doses. There was a notable drop in BCMA use during night shifts across all 15 wards. The local database highlighted that of 164,810 scannable medication encounters in the preceding 5 weeks, 68.7% were not scanned.

Three wards were identified for more detailed qualitative evaluation. On the three wards identified, scanning rates for medications were higher than those for patients. Our higher usage ward (orthopaedics) was scanning 39.4% of scannable medication doses, compared to a patient scan rate of 18%. This corresponded to rates of BCMA use on the lower usage ward (gastroenterology), where medication scan rates and patient scanning rates were 6.7% and 4% respectively. The mid-usage ward (urology/general surgery) demonstrated a 17% medication scan rate and 8% patient scan rate.

### Qualitative findings

#### Thematic analysis of nursing staff interviews

Ten nurses from the mid-usage ward were interviewed initially. These qualitative data were analysed iteratively, confirming the appropriateness of the topic guide and facilitating an initial identification of recurring themes. A further 17 nurses were interviewed (ten from the higher use ward and seven from the lower use ward), at which point thematic saturation was identified and recruitment ceased. Interviews were between 10 and 20 min in duration. Seventeen of the 27 sets of interview notes were coded by two members of the research group, with themes confirmed via ongoing discussion and conceptualisation within the wider study team. The thematic analysis led to the construction of a thematic framework housing 11 themes (Table 1). These themes reflected factors that potentially affected a nurse’s willingness, capacity and ability to use the BCMA technology. Qualitative data were continually re-reviewed and subthemes organised according to whether they reflected facilitators or barriers to BCMA. Of the 11 themes constructed within the thematic framework, five were heterogeneous and comprised data that reflected both facilitators and barriers. One theme purely concerned facilitators to BCMA use, and the remaining five comprised only barriers. Following triangulation of qualitative and quantitative data, qualitative themes corroborated the low scanning rates seen across all wards, and accounted for the variation seen across the wards (particularly related to patient acuity, staffing levels and the ward culture surrounding BCMA use).

**Table 1:** Thematic Framework incorporating facilitators and barriers to barcode medication administration (BCMA) use as identified in qualitative interviews with nursing staff

THEME	WORKING DEFINITION	SUBTHEMES	
		FACILITATORS	BARRIERS
BOTH FACILITATORS AND BARRIERS WITHIN THEMES			
TIME EFFICIENCY	Impact of BCMA on workflow, process and perceived impact on duration of drug round	- Streamlined Process - Perception of having time to use BCMA - Ability to stock appropriate drugs on trolley	- Viewed as time consuming - Difficulty scanning medications
CULTURE AND ACCOUNTABILITY	Presence of culture of using BCMA on ward, responsibility and potential repercussions	- Perception of BCMA as mandatory - Strong ward culture of BCMA - Enforced use - Encouraged use - View of BCMA as “standard practice”	- Lack of perceived repercussions - Not enforced
EQUIPMENT	Availability and usability of equipment required to perform BCMA	- Wireless scanners - Adequate numbers of scanners / trolleys	- Broken equipment - Software Issues
TRAINING	Provision of adequate training on BCMA system and ongoing support	- Thorough initial training - Ongoing support and troubleshooting - Informal support	- Delays between training and implementation - Lack of training for temporary staff
EFFECT ON PATIENT INTERACTIONS	Changes to interaction between nurse and patient when using BCMA for medication administration	- More time spent with patient at bedside - Patient aware of BCMA process - Patient has improved perception of safety	- Nursing focus on computer screen - Patient not receptive to wristband being scanned
ONLY FACILITATORS WITHIN THEME			
PATIENT SAFETY	Perceived safety benefits when using BCMA for medication administration	- Viewed by nurses as beneficial for patient safety - Improved nursing confidence that mistakes prevented	
ONLY BARRIERS WITHIN THEMES			
STAFFING AND WORKLOAD	Potential impact of staffing levels and workload on individuals perceived ability to use BCMA system		- High workload - Short-staffing - Role and Responsibilities
ERGONOMICS – BCMA TROLLEY	Impact of BCMA trolley design on ease of use and willingness to use		- Physical challenges of BCMA trolley - Unable to sit or write at trolley - Risk of injury
ERGONOMICS – WARD LAYOUT	Impact of ward layout on ease of using BCMA technology		- Obstacles on ward - Inconsistent wireless signal
INFECTION CONTROL	Implications of infection control measures on ability to use BCMA technology		- Uncertainty regarding policy and use of BCMA - Increased workload
DRUG RELATED	Limitations on use of BCMA technology resulting from prescriptions or drugs themselves		- Volume of medications to be scanned - Ability to scan medications

Barriers and facilitators were present on both high and lower usage wards, with individuals from both wards reporting that they viewed BCMA technology positively upon launch. Of note, there was consistent agreement among all participants that BCMA was perceived to be beneficial for patient safety.

#### Theme 1: time efficiency

The perceived impact of BCMA technology on time efficiency was heterogeneous across participants. Those on the higher use ward stated that they felt using BCMA helped streamline the process of administering medications (through stocking relevant medications within the new BCMA trolleys and reducing walking to and from the treatment room) and improved their ability to administer medications at the right time. However, the converse view that BCMA was in fact more time consuming (typically as a result of difficulty in scanning medications) was also present.

“streamlines the process, especially as medications are stored in the trolley” Nurse, higher use ward

“takes time to do so, not convenient for me to keep scanning patients and medicines as this is time consuming” Nurse, lower use ward

#### Theme 2: culture and accountability

A strong theme within the data set was the impact of ward culture and perception of accountability. Again subthemes were heterogeneous. The higher use ward seemed to have a strong culture promoting BCMA use (in which it was viewed as standard practice and encouraged). This contrasted with views from the lower use ward, with participants describing minimal repercussions for not using the system and a lack of culture promoting its use.

“incomprehensible that [we] wouldn’t use it” Nurse, higher use ward.

“Our manager enforces it so there are no issues with compliance” Nurse, higher use ward.

“Depends on the day – no overall culture of using it” Nurse, lower use ward.

“I don’t get in trouble if I don’t use it” Nurse, lower use ward.

#### Theme 3: equipment

Adequate numbers of functioning BCMA trolleys facilitated use of the technology, and many nurses reported that handheld wireless barcode scanners promoted ease of use. Again this theme was heterogeneous, with broken equipment, delays in repair, poor scanner connectivity and software anomalies reported as significant barriers.

“One BCMA trolley for each nurse/specific area so ‘no need to fight over computers’” Nurse, higher use ward

“sometimes the wired scanners don’t scan and there’s nothing we can do” Nurse, higher use ward.

#### Theme 4: training

Initial training was described positively by many participants, however there was some disparity regarding ongoing training and support. A lack of follow up, being unaware of how to access support and a delay between training and implementation of BCMA technology were cited as barriers.

“the training was very good…..two weeks after the training the team came to the ward and asked if we needed any help” Nurse, higher usage ward.

“On my night shift, I had no support on how to use it. There was a long time between my training and it being implemented.” Nurse, lower usage ward

#### Theme 5: effect on patient interactions

An unanticipated iterative theme that developed as interviews progressed was the perceived impact of BCMA on the quality of the interaction between the nurse and their patient. A variety of changes to this interaction were described. Some participants described how using BCMA allowed more time to be spent at the patient’s bedside and patient’s having a positive perception of improved patient safety. Barriers to BCMA use in the form of detrimental patient interactions were also reported, including the nurse’s focus being on the computer screen and patients not being receptive to having their wristband scanned.

“you get to engage with patients as you have to spend more time with them by the bedside” Nurse, higher usage ward

“you end up spending more time looking at a screen than the patient during the drug round” Nurse, lower usage ward

#### Theme 6: patient safety

A strong theme describing the perceived benefits of BCMA for patient safety and how this promoted BCMA use was present within the data. Participants from all wards described how using the technology improved their confidence that mistakes would be prevented, both in patient identification and administering the correct dose of medications.

“it is of benefit to patients because drug errors are minimised, you cannot overdose or underdose patients, you have to check the allergies, and you have to check the patient identity” Nurse, higher usage ward

“reducing drug errors benefits me and the patients, and ensures I give the correct medication” Nurse, lower usage ward

#### Theme 7: staffing and workload

The impact of staffing and workload was frequently described as a barrier to BCMA. The perception of either a high workload or inadequate staffing levels de-motivated individuals to use BCMA. A small sub-theme described the impact of an individual’s role – if they were acting in the role of nurse in charge, multiple competing interests and responsibilities led to an avoidance of BCMA.

“if we are short the nurse in charge has to join in on the drug round and they will usually do it without to save time” Nurse, lower usage ward

“In order for individuals to decide to use it needs to be fully staffed and have less patients” Nurse, lower usage ward

#### Theme 8: ergonomics – BCMA trolley

The design of the BCMA trolley (a newly introduced fixed height metal trolley housing space for medications and a mobile computer) created some barriers to use of the technology. Nurses on all wards described the ergonomics of the trolley as detrimental to facilitating its use. Physical challenges included being unable to adjust the height of the trolley, an inability to sit or write comfortably at the trolley and risk of injury when manoeuvring a stiff or heavy trolley around the ward.

“Legs have to be to side, cannot sit comfortably at trolley” Nurse, higher use ward.

“Cannot write without standing up” Nurse, lower use ward.

#### Theme 9: ergonomics – ward layout

The ward layout was described as creating barriers to BCMA, most notably through the presence of obstacles (typically medical equipment such as walking aids) or a lack of consistent connectivity in certain bays.

“I am unable to scan their medications due to the connection not reaching that area” Nurse, higher usage ward

#### Theme 10: infection control

Barriers arose as a consequence of uncertainty regarding the correct policy when utilising BCMA in infectious areas of the ward and through the creation of an increased workload caused by additional cleaning requirements.

“I am unsure about infection control and whether to take the trolley into the COVID bays or not” Nurse, lower usage ward

#### Theme 11: medication related

The final theme related to barriers arising from the medications themselves. This theme contained perspectives that linked to the workload theme, stating that high volumes of drugs required to be administered would reduce a participant’s likelihood to use BCMA. In addition, difficulty successfully scanning medications was also a barrier to BCMA use.

“less likely to use BCMA system if just giving one or two medications” Nurse, lower usage ward

“sometimes medicines don’t scan, have to do it manually” nurse, higher usage ward.

Further supporting qualitative data for these themes can be viewed in Appendix 4.

#### Supporting data from observations

Three drug round observations were undertaken. Data obtained through these observations predominantly related to the theme of “equipment”, providing more context to the impact of the BCMA technology on nurse’s willingness and capability to use BCMA in their daily work. Insights included a sense of “relief” in observed nurses if the BCMA scanner was not functional, and that many nurses exhibited frustration with the BCMA software – such as needing to frequently re-scan patients if they exited the software to review patient information. The wires on some scanners were observed as being short, leading to nursing dissatisfaction, particularly in the context of poor patient mobility. There was no observation in which all medications were able to be scanned, particularly when using patients’ own medication, linking to the theme of “drug-related” issues highlighted during nursing interviews.

#### Supporting data from patient interviews

Thirteen patients were interviewed (seven from the higher use ward and six from the lower use ward), at which point thematic saturation was deemed after review of the data by two researchers (KG and RH). Five had no knowledge or awareness of the BCMA system, with the remaining patients typically reporting that they observed BCMA being used “every time”. Of the 11 themes identified in the thematic framework, three themes were represented in patient interview data as factors that appeared to facilitate BCMA use (‘time-efficiency’, ‘patient safety’ and ‘equipment’), two themes were represented as factors that were seen as barriers to nurses using BCMA (‘drug-related’ and ‘training’), and two themes were present that contained perceived barriers and facilitators to BCMA (as experienced by nurses but observed by patients). These were ‘culture and accountability’ and the ‘effect on patient interactions. Patients viewed the system positively for their safety, feeling reassured that BCMA would help minimise the event of a drug error. Patients felt that the BCMA system was beneficial, efficient and did not observe some of the issues with the BCMA trolley ergonomics that nurses had reported. Contrasting some nursing views, patients felt that using the BCMA system was easier for staff at night as it caused less disruption, and they viewed the technology as making nurses’ “lives easier”. However, some patients did notice that a lack of training caused problems with BCMA use, and that occasionally they did not understand why they needed to be scanned repeatedly during their admission, when they had a rapport with the nurse looking after them. This increased understanding of patients’ experiences of BCMA was integrated with the conclusions drawn from the thematic analysis of nursing interview to provide greater context around the exhibited behaviours and providing more foci for future change initiatives. Supporting quotes from patient interviews can be viewed in Appendix 4.

#### Use of behavioural science frameworks to improve understanding of facilitators and barriers

Following detailed discussion and a conceptualised analysis within the study group, qualitative themes from the thematic framework mapped onto all components of COM-B and for both barriers and facilitators 11 of the 14 TDF domains (Tables 2 and 3).

This process highlighted several areas where behavioural change initiatives could be employed as part of a strategy to improve BCMA use, including an improvement of knowledge regarding BCMA, adoption of BCMA as part of an individual’s professional role and improving nurses’ belief in their capability to use BCMA technology within their working day.

**Table 2:** Facilitators to barcode medication administration (BCMA) use mapped to the COM-B (Capability, Opportunity, Motivation - Behaviour) Framework and Theoretical Domain Framework (TDF)

COM-B	THEORETICAL DOMAINS FRAMEWORK	THEMES (SUB-THEMES)
**CAPABILITY: PSYCHOLOGICAL**	KNOWLEDGE	**Training on BCMA** Awareness of BCMA system **Patient safety** Knowledge of links to patient safety
	MEMORY, ATTENTION AND DECISION PROCESSES	**Training on BCMA** Troubleshooting guidance available
**CAPABILITY: PHYSICAL**	SKILLS	**Training on BCMA** Provision of good skills-based training Ongoing support from training team / refresher sessions
**OPPORTUNITY: SOCIAL**	SOCIAL INFLUENCES	**Culture and Accountability** Culture of using BCMA on ward Encouraged to use by managers Encouragement and support from colleagues BCMA viewed as “standard practice” Perceived as mandatory Patients in support of BCMA system
**OPPORTUNITY: PHYSICAL**	ENVIRONMENTAL CONTEXT AND RESOURCES	**Equipment** Individual control over drugs on trolley Adequate number of scanners / trolleys for each drug round Wireless scanners **Time-efficiency** Reduced walking to treatment room
**MOTIVATION: AUTOMATIC**	REINFORCEMENT	**Culture and Accountability** Patients aware of BCMA system / expect use Organisational culture promoting / mandating BCMA
**MOTIVATION: REFLECTIVE**	PROFESSIONAL ROLE AND IDENTITY	**Culture and Accountability** Viewed as part of nursing role
	BELIEFS ABOUT CAPABILITIES	**Time-efficiency** Streamlined process / Time-saving **Training on BCMA** Adequate training and confidence using system **Patient safety** Belief that using system will improve patient safety Confidence regarding avoidance of drug error provided by BCMA system
	GOALS	**Patient Safety** Minimise drug errors **Effect on patient interactions** More time spent by patient’s bedside
	BELIEFS ABOUT CONSEQUENCES	**Patient Safety** Confidence / reassurance from using BCMA system **Culture and Accountability** Organisational culture mandating BCMA
	OPTIMISM	**Patient Safety** Confidence that patient safety will be improved

*TDF domains not mapped to qualitative data on facilitators were “behavioural regulation”, “emotion” and “intentions”

**Table 3:** Barriers to barcode medication administration (BCMA) use mapped to the COM-B (Capability, Opportunity, Motivation - Behaviour) Framework and the Theoretical Domain Framework (TDF)

COM-B	THEORETICAL DOMAINS FRAMEWORK	THEMES *(SUB-THEMES)*
**CAPABILITY: PSYCHOLOGICAL**	KNOWLEDGE	**Training on BCMA** Unaware of troubleshooting guides **Infection Control** Uncertainty regarding using BCMA
	MEMORY, ATTENTION AND DECISION PROCESSES	**Training on BCMA** Delays between training and implementation **Time-efficiency** Lack of cognitive capacity to use system with available staffing and workload
**CAPABILITY: PHYSICAL**	SKILLS	**Training on BCMA** Lack of follow up / refresher sessions Inadequate training Temporary staff not trained
**OPPORTUNITY: SOCIAL**	SOCIAL INFLUENCES	**Culture and Accountability** No ward culture of using system **Effect on patient interaction** Patients not receptive to wristband being scanned.
**OPPORTUNITY: PHYSICAL**	ENVIRONMENTAL CONTEXT AND RESOURCES	**Staffing and Workload** Short-staffing reduces perceived feasibility High workload limits use of BCMA **Ergonomics of BCMA Trolley** Physical challenges when using BCMA trolley **Ergonomics of ward** Physical Obstacles Wireless signal not consistent **Drug-Related** Unable to scan some medications Number of Medications **Equipment** Hardware malfunction Software issues
**MOTIVATION: AUTOMATIC**	REINFORCEMENT	**Culture and Accountability** Use not enforced out of hours Lack of perceived repercussions Not enforced by management **Effect on patient interaction** Diminished patient interaction
**MOTIVATION: REFLECTIVE**	PROFESSIONAL ROLE AND IDENTITY	**Workload / Staffing** Other competing responsibilities as nurse in charge
	BELIEFS ABOUT CAPABILITIES	**Time-efficiency** Perception that don’t have enough time to use BCMA
	GOALS	**Workload / Staffing** Requirement to complete other tasks
	INTENTIONS	**Time-efficiency** Perception that BCMA takes longer
	BELIEFS ABOUT CONSEQUENCES	**Culture and Accountability** Lack of perceived repercussions

*TDF domains not mapped to qualitative data on barriers were “behavioural regulation”, “emotion” and “optimism”

## Discussion

We present a detailed analysis of the facilitators and barriers to the use of BCMA using data from qualitative interviews and observations to explore and explain quantitative findings. The robustness of this analysis is enhanced by the inclusion of three wards with differing levels of BCMA usage. Uniquely, we captured data encompassing the patient’s perspective, providing a more holistic understanding of how BCMA technology can affect those involved in medication administration, and potentially affect interactions between patients and nursing staff. Themes that originated during our qualitative analysis were then integrated with quantitative data to further understand patterns which were seen. These included a lower scan rate a night, and different rates between wards. Notably, we highlighted that a lower scan rate was associated with a less present “culture” of using BCMA, and less accountability for individuals associated with its use.

Mapping the findings of the thematic analysis to established behavioural science frameworks is a novel method of utilising qualitative data obtained regarding the facilitators and barriers to BCMA use. This was successful in furthering our understanding of the origins of existing behaviour, and provides a basis for the design of future behavioural science informed interventions aimed at improving scanning rates.

The barriers to BCMA identified within the thematic framework corroborate those identified in existing literature, including a perception that using BCMA was lengthier than previous practice and difficulties with scanning certain medications [6, 12, 24, 25]. A less represented theme in previous literature was strongly present within our analysis; physical difficulties associated with the ergonomics of the newly introduced BCMA trolley.

Interestingly, the barriers to BCMA usage (including ergonomics of the BCMA trolley and difficulty scanning medications) were present across all three wards. This suggests that there is a significant behavioural component in nurses feeling empowered to overcome such barriers and continue using BCMA on some wards. The presence of barriers to BCMA use can lead to the adoption of workarounds – thereby avoiding the BCMA system yet still administering patient medications. Such workarounds include not scanning the patient / medication and avoiding secondary safety checks [24]. It has been shown that typically these workarounds are developed within the first 24 h of using the technology [13]. A qualitative study using focus groups found that 50% of participants used a workaround to the BCMA system in their previous shift, in general arising as a consequence of technology failure (including inability to scan) or a perception that the system is too time consuming [26]. Our data corroborate the behaviour seen in these previous studies – as many nurses described using the original system (that did not require medication to be scanned, and still in place to allow for “unscannable medications” to be administered) due to the barriers identified in our analysis, such as short staffing, feeling time-pressured, or the ergonomics of the BCMA trolleys. Understanding the behaviours that result from such barriers can lead to thoughtful interventions that will enhance an individual’s capability to use systems such as BCMA, thereby avoiding workarounds becoming an “easier” or more straightforward option.

Whilst many published studies focus purely upon the barriers to BCMA use, our qualitative analysis yielded a large number of facilitators. These included the presence of a palpable culture of using BCMA, leadership encouraging and enforcing BCMA use, feeling confident and reassured by using BCMA (in terms of medications safety) and having received adequate and ongoing training. The importance of these themes were highlighted within the use of behavioural science frameworks. This knowledge of both facilitators and barriers to BCMA enhance the understanding of the behaviours associated with BCMA use, and provide a good basis from which to design interventions and create change.

### Limitations

This work has several limitations. Whilst a broad range of nurses were purposively sampled (both in terms of BCMA experience and level of seniority), it is possible that some perspectives were not included – those with no interest in using the BCMA system may not have been inclined to participate in an interview; equally those who used it regularly may not have been motivated to discuss it further. Temporary and agency staff were not interviewed, again risking missing a different viewpoint on BCMA.

A range of patients were interviewed, however those who were very unwell or had communication difficulties were not suitable for inclusion. Whilst the viewpoints of patients interviewed regarding BCMA were generally positive, we may therefore have missed some patient experiences and perspectives.

A further limitation is that the interviews (both with nursing staff and patients) and observations were short in duration, and a limited number of observations were conducted. This reflected the time available for interviews given clinical commitments in the busy ward environment, and a desire not to over-burden patients. However, it may be that conducting longer interviews would have provided a richer data set, and more detail within each theme.

The use of behavioural science frameworks to further understand the behaviours associated with the barriers and facilitators to BCMA use is novel, and provides a helpful way of organising qualitative data so that it can be used to guide behavioural science informed interventions. However, the interview topic guide was designed to elicit facilitators and barriers rather than exploring each component of these behavioural science frameworks. As such, there may have been specific behaviours that were not explored, which may explain why only our data represented only 11 of the 14 TDF domains. Future studies could be designed using behavioural science frameworks in a more deductive manner, ensuring all TDF domains are investigated.

This was a service evaluation project within one UK NHS trust, and as such it was not designed to be generalisable. However, wards with similar constraints, staffing models and patient cohorts may benefit from the learning within this project when implementing BCMA in their own clinical environments, as barriers and facilitators may be similar.

## Conclusion

This study presents a novel exploration of the experiences of BCMA technology in both nursing staff and patients, with an overwhelming perception across both participant groups that BCMA is beneficial for patient safety. This study utilised an explanatory mixed methods approach to obtain an in depth and detailed understanding of the facilitators and barriers to BCMA use on wards with high and low usage. Barriers and facilitators were present across all wards, emphasising the importance of motivations and behaviours in adopting new technologies. Of particular significance was the importance of a strong ward culture and accountability in motivating individuals to use BCMA. The study incorporated patient and nursing interviews to obtain a more holistic understanding of how BCMA technology has been adopted within the clinical environment, the challenges to adoption and how BCMA technology is perceived. Applying qualitative data to behavioural science frameworks provided an increased understanding of how these behaviours manifest, with 11 of 14 domains on the theoretical domains framework represented in our thematic analysis. This provided insight into the influences on nursing behaviour with patient safety a prominent motivator. The use of the COM-B framework highlighted areas of capability, opportunity and motivation within the observed and described behaviours that can act as foci for future interventions aimed at creating behaviour change.

Reassuringly, all nursing participants emphasised the benefits of BCMA for safety and confidence in their medication administration rounds. The use of wards with different scanning rates allowed barriers and facilitators to be identified, but also provided a sense of which barriers were modifiable and might be amenable as a point for behavioural science informed intervention design.

### Electronic supplementary material

Below is the link to the electronic supplementary material.

Supplementary Material 1

Supplementary Material 2

Supplementary Material 3

Supplementary Material 4

## Data Availability

All data generated or analysed during this study are included in this published article and its supplementary files. Original interview notes analysed during the current study are available from the corresponding author on reasonable request.
