# Supplementary material for: Understanding the facilitators and barriers to barcode medication administration by nursing staff using behavioural science frameworks. A mixed methods study
Source: BMC Nurs. 2023 Oct 12;22:378. doi: 10.1186/s12912-023-01382-x (PMC10571469; doi:10.1186/s12912-023-01382-x)
Supplement: Supplementary file 1 — Supplementary Material 1 [file 12912_2023_1382_MOESM1_ESM.docx]

**Appendix 1: Topic Guide for Semi-Structured Interviews- Nurses**

Introduction with brief overview of topic

Example introduction: “We are a team from the Change Lab at Imperial College London, and we are evaluating the use of barcode scanning of medicines administration at Imperial on wards where it is now being used.

The purpose of this interview today is to improve our understanding of your experiences with BCMA by asking you a few questions. This should take no longer than 10 – 20 minutes, and you are free to leave the conversation at any point.”

*Confirm likely availability for duration of conversation and consider asking if a quieter location is available if appropriate.

“I may take notes during our conversation, but these will not be stored with any information that might identify you. The purpose of the notes is to allow us to reflect back on our data and occasionally use quotes in any reports or publications, but you will not be identifiable from these.”

“Do you have any questions for me about this project?”

“Are you happy to proceed with the interview and are you happy to consent to today’s interview?”

**General/opening**

“Can you tell me your staff role, and if you have experience of using BCMA?”

(Make note of seniority of staff member – junior (band 5/6) or senior)

Q1. Could you describe your experience of using BCMA?

a. What are positive aspects?

b. And negative?

Q2. Did you experience any difficulties when you first started using BCMA, or were first asked to use it?

- Elaborate on these difficulties as appropriate

a. If not using BCMA at all can explore why here

Q3. What helped you/would help you in using the BCMA system more often?

Q4. Do you feel BCMA is of benefit to your patients’?

- Can expand here about whether participants understanding the safety benefits

Q5. Have you noticed any unintended consequences or negative outcomes as a result of using this new system?

Q6. How do you think the patients view this new system?

**Identification of specific barriers/facilitators (not yet discussed)**

“I will now ask you a few questions about a few other potential factors that we have not yet discussed.”

**Training**

Q7. Did you receive any training or guidance prior to use? Did you feel this adequately supported you in using BCMA independently?

a. If so, what aspects of the training were particularly helpful?

b. If not, what aspects of the training can be improved to better support staff such as yourself?

Q8. Do you feel that you have the ongoing support you need to use BMCA after this initial training (if received)

Q9. Has it been easy to adopt the change in practice and use of new technology required?

**Working environment**

Q10. Are there any issues within this workplace or equipment (such as poor WiFI or broken scanners) that lead to problems using the BCMA system

Q11. Does the ward layout make it easier or harder for you to use this system?

Q12. What equipment or additional resources would better enable you to use the BCMA system?

Q13. We have noticed in our initial data review that there are differences in the use of BCMA across wards and staff groups. Do you feel there is a culture of using BCMA on this ward?

Q14. Is this impacted by staffing levels?

- As appropriate can explore leadership culture / support

- Could expand upon how this influences the participants use

**Structure of day**

Q15. We have found that BCMA is sometimes used more often during day shifts compared to night shifts. What do you think could be the reasons for this?

**Medication route**

Q16. Do you think the BCMA system is useful for all types of drugs? Why/why not?

Q17. Are there any other drugs that you wish you could scan?

**Close**

Q18. Is there anything else you’d like to add that we haven’t discussed already?

Wrap up interview and thank participant. Offer opportunity to ask any questions.
