# Supplementary material for: Understanding the facilitators and barriers to barcode medication administration by nursing staff using behavioural science frameworks. A mixed methods study
Source: BMC Nurs. 2023 Oct 12;22:378. doi: 10.1186/s12912-023-01382-x (PMC10571469; doi:10.1186/s12912-023-01382-x)
Supplement: Supplementary file 2 — Supplementary Material 2 [file 12912_2023_1382_MOESM2_ESM.docx]

**Appendix 2: Topic Guide for Semi-Structured Interviews - Patients**

Introduction with brief overview of topic

Example introduction: “We are part of a research team at this hospital, and we are evaluating the use of barcode scanning of patients and medicines administration on wards where it is now being used.

We would like to improve our understanding of patients’ experiences with barcode medication administration. Are you happy for me to ask you a few questions? This should take no longer than 5-10 minutes, and if you feel tired or for any other reason, you are free to ask to end our conversation at any point. Whether or not you participate in the interview, this will have no effect on your medical care.

I may take notes during our conversation, but these will not be stored with any information that might identify you. The purpose of the notes is to allow us to reflect back on our data. We might use quotes in any reports, but you will not be identifiable from these. I am not recording your name or any other information that might identify you.”

“Do you have any questions for me about this project?”

“Are you happy to proceed with the interview and are you happy to consent to today’s interview?”

**General/opening**

1. How long have you been in hospital so far?
2. Are you aware of the system for barcode scanning of medicines in use on this ward?
3. What is your understanding of the system? Do you know what the system does?

[prompt if seems quite knowledgeable about the system: did a member of staff explain to you what the system does?]

**Experience with bar code medication administration**

1. If patient aware of BCMA:

“What is your experience with this system: 
-What are the positive aspects? 
-What are the negative aspects?”

[If they refer to previous systems / previous hospital stays, need to clarify what they are comparing to].

1. If patient not aware:

“how would they feel about nurses scanning their wristband to confirm their identity before giving them their medications?”

**Patient’s view on bar code medication administration**

1. What effect do you feel this system has on the nursing staff?
2. Do you think the barcode scanner is more helpful at particular times of the day?

**Close**

1. Do you have any other comments? Anything we’ve not covered that you would like to add?

Wrap up interview and thank participant. Offer opportunity to ask any questions.
