# Supplementary material for: Understanding the facilitators and barriers to barcode medication administration by nursing staff using behavioural science frameworks. A mixed methods study
Source: BMC Nurs. 2023 Oct 12;22:378. doi: 10.1186/s12912-023-01382-x (PMC10571469; doi:10.1186/s12912-023-01382-x)
Supplement: Supplementary file 4 — Supplementary Material 4 [file 12912_2023_1382_MOESM4_ESM.docx]

Appendix 4 – Supporting Qualitative Data from Nursing and Patient Interviews

Inductive Thematic Analysis of Nursing Interview Data

| THEME | SUB-THEMES | SUPPORTING DATA |
| --- | --- | --- |
| **FACILITATORS** | **Time-efficiency**   - Streamlined process - Reduced walking to treatment room - All drugs on trolley - Can stock relevant medications on trolley | “same work but with increased peace of mind”  “having all medications in trolley minimises need to walk back and forth across ward”  “nurses stock trolley – viewed positively as can ensure what you need is on there”  “streamlines the process, especially as medications are stored in the trolley”  “found new system easier to use”  “easier to have medications on the trolley and its easier when using ward stock medications, because everything is placed in the BCMA trolley”  “we can give the patients their medications more promptly/on time”  “easy to use, all medicines are available in the trolley”  “majority of the medicines are in the treatment room so you don’t need to go back and forth”  “ensure we are scanning right medicine and right patient” |
|  | **Culture and Accountability**   - Sense of it being mandatory - Enforced by management - Room for improvement - Required encouragement at first | All nurses reported to be using on ward  “no option not to use system”  “incomprehensible that wouldn’t use it”  Very good culture of using BCMA  Could be improved by reminders – in handover  “I don’t have the choice so I have to use it every time I prescribe drugs”  “Our manager enforces it so there are no issues with compliance’  “everyone does use it during the drug round”  “Most medicines are now given by BCMA, its now normal”  “we received an email from our matron to always use this” |
|  | **Patient Safety**   - Improves confidence that won’t make a mistake - Seen as being beneficial for patient safety - Dose corrections | “gives me confidence”  “good for patient safety – prompts dose corrections and minimises risks of mistakes”  “patient identification much better”  “less error, much safer for patients”  “reduced drug errors because you’re double/triple checking”  “I feel as if patients trust me more”  “I think when scanning medications, its more accurate and can potentially reduce drug errors”  “it improves safety because if you miscalculate dose, the system does not go ahead”  “it is of benefit to patients because drug errors are minimised, you cannot overdose or underdose patients, you have to check the allergies, and you have to check the patient identity”  “it makes you scan twice if you are giving two tablets which is helpful so you don’t forget to give a second one”  “quicker and reduces drug errors”  “reducing drug errors benefits me and the patients, and ensures I give the correct medication”  “this would also reduce drug errors, because you’re scanning the actual medicine”  “this can also reduce medication errors”  “yes, useful in avoiding drug errors”  “being able to scan the patient’s wristband and then all their medicines appear, so you know it’s the right patient and right medicines for that patient”  “right patient and reduces drug errors”  “it confirms patient identity, makes sure patient is wearing a wrist band with a barcode and ensure the patient matches the medications they are on”  “good way to make sure correct medicine is given at the right time”  “if you scan the wrong medicine it will say its wrong”  “if you need to give 10mg of prednisolone and you only scan 5mg, you cannot move onto the next medicine until you scan twice”  “reduces drug errors, ensures it’s the correct patient by scanning patient and ID band”  “it ensures we scan the right person”  “Matches patient identification, so we know its the correct patient”  “makes sure the right medicine is administered for the right patient” |
|  | **Equipment**   - Much easier with wireless scanner - Plenty of BCMA trolleys on ward | “wireless is perfect”  Wireless makes logistics easier  Wireless better for infection control, reduced need to push trolley around  “Wireless reduces awkwardness of getting to patients wristband”  One BCMA trolley for each nurse/specific area so “no need to fight over computers”  “straightforward to use, no need to scroll down on screen”  “all wireless scanners would be useful, as wired scanners are too short”  “it could be slightly easier if all the scanners were wireless, but its not a huge issue”  “we have one scanner per nurse” |
|  | **Training**   - Good training provided initially - Ongoing support available - Support from colleagues | “the training was very good…..two weeks after the training the team came to the ward and asked if we needed any help”  “I do feel like I have ongoing support and if I do get stuck on anything I know who to ask”  “The training was intense and covered everything”  “The team were always available within hours”  “yes I can call them if I need any help”  “training was good, just needed practice and encouraging everyone to use it”  “the training was very engaging”  “I don’t need further support but I know who to contact if I need to”  “training was good”  “I do feel like I have ongoing support”  “they would come back to the ward to visit and ask if we needed any help”  “all of the training was helpful in preventing accidents”  “We were able to ask questions and the demo was helpful”  “training was informative and I felt supported’ |
|  | **Effect on patient interactions**   - Can improve patients perception of safety - No impact on interaction - Heterogenous views on this theme | “you get to engage with patients as you have to spend more time with them by the bedside”  “patients sometimes pick up that you haven’t scanned their wristband”  “I think patients view this system as being safer”  “patients think it’s a new technology and is good”  “patients never complain”  “feels that patients are more confident and it gives them ‘peace of mind’” |
| **BARRIERS** | **Staffing and Workload**   - Heterogenous views - Links to time consuming subtheme - NIC   - Competing interests   - Lack of TIme | “In order for individuals to decide to use it needs to be fully staffed and have less patients”  “nurses will still use BCMA even if its busy”  “we still use it even if short staffed”  “if short staffed then less likely to use, especially because our ward is almost always full”  “100% if short staffed then we wouldn’t use BCMA, especially for the morning medications as it would take a lot of time”  “Takes longer to scan”  At night: “staffing levels [inhibit use] as its an inconvenience when we are short of staff due to it being so time consuming”  “on this ward there’s around 6-8 patients per nurse, so its a lot busier”  “if we are short the nurse in charge has to join in on the drug round and they will usually do it without to save time”  “i dont use the scanner all the time, initially I tried to be compliant, however its difficult on this ward especially the 8am drug round is so busy” |
|  |  |  |
|  | **Ergonomics of BCMA Trolley**   - Unable to sit at trolley - Unable to write notes at trolley - Requirement for two trolleys - Less likely to switch trolley for 1 off drug administration - Unable to adjust height of trolley | “would be improved if can adjust height of trolley”  “very challenging physically to stand on feet for 12 hours”  “very uncomfortable”  Legs have to be to side, cannot sit comfortably at trolley  Cannot write without standing up  “Trolleys need good steer – can be stiff and need to pull trolley – risking injury”  “The door code on the drug trolley itself is so annoying to use – its not ergonomic”  “the medicines trolley is heavy and hurts my back, I don’t feel safe when pushing the trolley as it’s quite big so blocks my view in front”  “the trolley is heavy and hurts my back, and the monitor is quite big too”  “the cord is very short and the trolley is heavy when trying to wheel..to patients”  “The height of the computer is an issue, when sitting down, the height of the computer is too high, this hurts my back” |
|  | **Ergonomics of ward**   - Awkward especially if wired scanner - Patient equipment in way - Obstacles on ward | Obstacles: Patient equipment, small space between beds  “the doors to enter the ward bays are very small, so its difficult to get in and out of the bays with the BCMA trolley” |
|  | **Time-efficiency**   - Time to scan patient - Time to scan each drug (esp if drugs don’t scan) | “too much time taken scanning each medicine, particularly if short staffed”  Some boxes cannot be scanned – leading to “override of system”  “Unable to scan white boxes provided by Pharmacy”  “sometimes medicines don’t scan, have to do it manually”  “because we have to scan one by one and it takes time, especially at 8am, patients will have the most medications and it takes longer to administer with BCMA”  “takes time to do so, not convenient for me to keep scanning patients and medicines as this is time consuming”  “I would still prefer manual scanning, I think BCMA is more time-consuming”  “time consuming to scan” |
|  | **Culture and Accountability**   - Repercussions - Not enforced during night shifts | “I don’t get in trouble if I don’t use it”  No repercussions for not using  “Depends on the day – no overall culture of using it”  “no one there to check at night”  “People are lazier on night shifts and there is no manager to keep an eye”  “You can get around it by pressing next and cheating so you don’t actually have to scan anything”  “nurse in charge on this ward has said we don’t need to use BCMA, whereas on other wards they might have to use it regardless”  “I am more against using it, I prefer the old way of manually scanning and removing medicines from medicine cupboard”  “Nurse in charge said we do not need to use BCMA”  “I always use it, but my colleagues don’t, because they said scanning takes time” |
|  | **Infection control**   - Difficulty taking trolley into infectious areas - Improved with wireless scanner | Wireless scanners much better for COVID/infectious bays – but often not enough on ward  Lack of clarity regarding taking BCMA trolleys into covid bays – “if we keep the trolleys outside its difficult to keep going back and forth to the patient’s bed and trolley”  “I am unsure about infection control and whether to take the trolley into the covid bays or not” |
|  | **Equipment**   - Broken screens - Wifi signal - More difficult with wired scanners - Need to remember to charge computer | “if machine battery flat and system shuts down takes 30 minutes to recharge”  “one screen cracked – still not fixed”  “must remember to charge machine”  “doesn’t get rid of old dates – shows entire admission, risk of clicking wrong date, system will let you scan and add meds to a previous date”.  “drug chart shows medications from day before”  “Sometimes signal is poor and scanners don’t work”  Software very “glitchy”  “sometimes the wired scanners don’t scan and there’s nothing we can do”  “sometimes it works, sometimes it doesn’t”  “wireless scanners can take a while to configure. If a computer has been left alone for a day, then when I restart it it takes longer to set up”  “The battery life of the computers isn’t great”  “it can go out of range, especially with the wireless scanners”  “i am unable to scan their medications due to the connection not reaching that area” |
|  | **Effect on Patient Interaction**   - Can be detrimental to interaction - Patient perception of system | “sometimes patients have said ‘am I a supermarket’ because we scan them”  “it is difficult with patients that are confused/don’t speak English”  “you end up spending more time looking at a screen than the patient during the drug round”  “I don’t like to disturb patients at night, I don’t like to put the lights - on and disturb the patient by scanning their wristband”  “8am medication round is busy and takes longer to administer medications with BCMA”  “older patients think it is very high tech”  “nurses attention on screen rather than patient” |
|  | **Drug-related**   - Ability to scan medication - Volume & complexity of drugs per patient | “More useful if we could scan all drugs”  “less likely to use BCMA system if just giving one or two medications”  “I would like to be able to scan IV medicines and Insulin as this would be safer”  “I would prefer to scan IV drugs, it would be more clear if there was a process” |
|  | **Training**   - Lack of follow up - Implementation - Temporary staff | “training was quite quick and short, maybe it could’ve been longer”  “I didn’t receive any follow up or refresher training, would have been useful to have”  “we don’t have time to train agency staff how to use the barcode scanner”  “at the start we were rushed to start using it”  “I felt like we were audited straightaway and people were asking why we weren’t using it”  “not sure how to troubleshoot if problems arise”  “training was way before it came into effect so I had forgotten how to use BCMA by the time it was implemented”  “On my night shift, I had no support on how to use it. There was a long time between my training and it being implemented.” |

Deductive Thematic Analysis of Patient Interview Data

| THEME | SUB THEMES | SUPPORTING DATA |
| --- | --- | --- |
| **FACILITATORS** | **Time-efficiency** | “Nurses can simply scan at night-time and doesn’t have to disturb me up to provide identity information”  “Easier for nursing staff, less laborious, “taking a load off the team.”  “Easier for nursing”  “Always use BCMA and scan before administering medication”  “Perceives system as being easier for nurses”  “Especially good at night, reducing patient disturbance.” |
|  | **Culture and Accountability** | “BCMA is always used prior to medication administration.”  “Always being used, every single time” |
|  | **Patient Safety** | “Used to identify the patient with the right medicine and right dose.”  “Sounds like a good system, because it ensures everything matches up.”  “Reassured that “no room for drug error.”  “Patient feels safer with BCMA being used and less risk of drug error”  “Reduced human factors and helps reduce medication errors.”  “Good idea and reduces drug errors.”  “Helps nurses in reducing drug errors.” |
|  | **Equipment** | “Great improvement in technology”  “System appears up to date”  “Seems to be much quicker”  “System is very efficient”  “Plenty of space for trolleys”  “No observed issues using BCMA trolley” |
|  | **Effect on Patient Interactions** | “Very beneficial”  “Beneficial for patient and no concerns identified”  “Did not observe any problems for nurses when using the system.”  “Can’t fault them”  “Happy with the nurses service”  “Don’t mind being scanned, the system feels safer to use.” |
| **BARRIERS** | **Culture and Accountability** | “Not observed nurses scanning wristbands prior to being given medications.”  “The nurses have not been scanning my wristband and medications.”  “Unaware of the BCMA system as I have not seen it being used.” |
|  | **Effect on Patient Interactions** | “Doesn’t understand why nurses need to ask patients to confirm name and date of birth every time they administer medications.” “I”m the same patient and the nurses recognise me, so is there a need to keep asking?”  “Not sure why nurses are scanning my wristband”  “Was in too much pain at night time to notice if my wrist was being scanned prior to administration.” |
|  | **Drug-Related** | “My own medicines can’t be scanned, it would be quicker if they could be scanned.” |
|  | **Training** | “Occasionally junior/ new staff do not have correct access to system/cerner card but are required to use it. Logistically this can be an issue.” |
